# The History and Capabilities of Herbal Simples

**Published:** 1894-01-27

**Authors:** W. T. Fernie


### THE HISTORY AND CAPABILITIES OF HERBAL SIMPLES.

By W. T. Fernie, M.D. *(Continued from page 420, Vol. XIV.)*

### GOOSE-GRASS.

"Goosey! goosey! gander! whither do ye wander?" says an old nursery rhyme, by way of warning to the silly waddling birds not to venture into hedgerows, else will they become helplessly fettered by the tough, straggling coils of clivers, goose-grass, or hedge-heriff freely growing there, and a sad despoiler of feathers. This herb, the Galium aparine, which is a highly useful curative simple, springs up plentifully about fields and waste places in most English districts. It belongs to the rubeaceous order of plants, all of which have a root like the madder, affording a red dye. The hardy goose-grass climbs courageously by its slender, hairy stems through the dense vegetation of our hedges into open daylight, having sharp serrated leaves, and bearing small white flowers "pearking on the tops of the sprigs." The herb is also called Cleavers, because cleaving to the clothes of passers-by; likewise "love-man," from its clinging closely to people; and beggars' lice, from the same reason, and because the small burs resemble these vermin. Furthermore, it is known as catchweed, tongue-bleed, burweed, and cliders; also as grip-grass, from its gripping or seizing with its small recurved bristles whatever comes in its way. Its botanical adjective, "aparine," bears the same meaning, being derived from the Greek verb apairo, to lay hold of. And the generic title Galium, comes from the Greek word gala (milk), which the herb was formerly employed to curdle instead of rennet. With the well-known little Miss Muffet of our childhood's days it must therefore have found particular flavour. That misguided young lady was of sedentary habits, and ate to repletion of curds and whey. Need I say she soon suffered from nervous indigestion?

There came a great spider, which sat down beside her,
And frightened Miss Muffet away.

Evidently her nerves were nowhere, and plain food with plenty of exercise would have made her far more plucky. The appellation cheese-rennet, or cheese-running—from gerinnen, to coagulate—has been given for the above reason to this plant and to other bed-straws; whilst children, again, call its leaves whip-tongue, and use them in play to draw blood from their tongues. Also this goose-grass is sometimes named goose-bill—from its thin, long, bristly leaves being like the rough-edged mandibles of a goose,—or goose-share, it being a certain fact that goslings are extremely fond of the plant. Country persons often take it in spring broth to purify the blood. The Galium aparine (goose-grass or cleavers) has a special reputation for curative uses when employed against cancerous diseases and tumours. For open cancers an ointment is made from the whole plant for dressing the ulcerated surfaces, and at the same time the expressed juice is given internally. Dr. Tuthill Massy declares "it often effects a cure in from six to twelve months; and it should be drank regularly afterwards in the springtime." Dr. Thornton, in his excellent "Herbal" (1810), says after some eminent surgeons had failed, he ordered the juice mixed with linseed to be applied to the breast, with a teaspoonful of the same juice to be taken every night and each morning while fasting; by which plan after a short time he dispersed very frightful indolent tumours in the breast. On chemical analysis the herb is found to contain three distinct acids—tannic acid (of galls), citric acid (of lemons), and a special rubichloric acid. Dr. Quinlan, at St. Vincent's Hospital, Dublin, successfully used the fresh juice applied as a poultice three times daily to chronic ulcers of the legs. Its effect, he says, in the most unhopeful cases was decisive and plain. The concentrated fresh juice, and a decoction of the plant for lotions, and an ointment for cancerous sores are now trustworthily made by some of our leading druggists. Or, the plant may be very readily got in the summer from most green hedges in our country districts. In cancer four or five fluid ounces of the freshly-expressed juice should be taken twice a day. Its thin, square, trailing stems, rough at their edges, with leaves arranged in whorls around the stalks, and with the other characteristics already given sufficiently distinguish this goose-grass, which when bruised has a somewhat acid taste, and will arrest bleeding from open wounds or sores. Dioscorides said, in his day, "Shepherds do use the plant to take hairs out of milk, if any remain therein." And old Gerard says, "The herb stamped with swine's grease wasteth away kernels by the throat. Women also do commonly make pottage of cleavers with a little mutton and oatmeal to cause leanness, and to keep them from fatness." The flowers bloom towards the end of August, about the time of the Feast of the Annunciation, and they are supposed to have first burst into blossom at the birth of our Saviour. Irish peasant girls frequently employ the round seeds wherewith they repeat their "aves," using them for a rosary in place of beads. Some suppose that the goose-grass and other galiums are hence called bed-straws, or bead-straws. These include the common yellow bedstraw, which is abundant on dry banks, chiefly near the sea, being known by its small puffy stems, and small golden flowers closely clustered together in dense panicles. An ointment (says Gerard) made from its flowers is good for anointing the weary traveller. These plants are dedicated to Our Lady, the Virgin Mary. Most probably their title "Bed-straw" refers to the fact that straw was formerly employed for bedding, even by ladies of high rank—whence came the expression of their being "in the straw" at interesting times of married life. The word "straw" comes from the Latin verb, sterno—to strew, or scatter about. Highlanders make special use of the yellow bed-straw for colouring cheese, and for curdling milk. This plant because of its bright yellow blossoms is also named maid's hair, like the loose unsnooded golden hair of maidens. In Henry VIII.'s reign "Maydens did wear silken callis to keep in order their hayre made yellow with dye." For a similar reason the yellow bed-straw is known too as Petty Mugget, from the French *petit muguet*, a little dandy, applied in ridicule to effeminate young men, the Jemmy Jessamies, or mashers of the period. *Par excellence*, it is Our Lady's bedstraw, who gave birth to her Son in a stable with nothing but wild flowers for her bedding. Thus in an old Latin hymn she sings very sweetly:—

Lectum stravi tibi soli; dormi, nate bellule!
Stravi lectum fœno molli; dormi, mi animule!
Ne quid desit sternam rosis, sternam fœnum violis;
Pavimentum hyacinthis, et prœsepe liliis.
Sleep, sweet little babe, on the bed I have spread thee!
Sleep, fond little life, on its straw scattered o'er!
'Mid the petals of roses and pansies I've laid thee
In crib of while lilies—blue-bells on the floor.

